# Cold-induction of afadin in brown fat supports its thermogenic capacity

**DOI:** 10.1038/s41598-021-89207-2

**Published:** 2021-05-07

**Authors:** Morten Lundh, Ali Altıntaş, Marco Tozzi, Odile Fabre, Tao Ma, Farnaz Shamsi, Zachary Gerhart-Hines, Romain Barrès, Yu-Hua Tseng, Brice Emanuelli

**Affiliations:** 1 Novo Nordisk Foundation Center for Basic Metabolic Research, Faculty of Health and Medical Sciences, University of Copenhagen, Copenhagen, Denmark; 2 Joslin Diabetes Center, Harvard Medical School, Boston, MA USA

**Keywords:** Physiology, Endocrinology

## Abstract

The profound energy-expending nature of brown adipose tissue (BAT) thermogenesis makes it an attractive target tissue to combat obesity-associated metabolic disorders. While cold exposure is the strongest inducer of BAT activity, the temporal mechanisms tuning BAT adaptation during this activation process are incompletely understood. Here we show that the scaffold protein Afadin is dynamically regulated by cold in BAT, and participates in cold acclimation. Cold exposure acutely increases Afadin protein levels and its phosphorylation in BAT. Knockdown of Afadin in brown pre-adipocytes does not alter adipogenesis but restricts β_3_-adrenegic induction of thermogenic genes expression and HSL phosphorylation in mature brown adipocytes. Consistent with a defect in thermogenesis, an impaired cold tolerance was observed in fat-specific Afadin knockout mice. However, while Afadin depletion led to reduced *Ucp1* mRNA induction by cold, stimulation of Ucp1 protein was conserved. Transcriptomic analysis revealed that fat-specific ablation of Afadin led to decreased functional enrichment of gene sets controlling essential metabolic functions at thermoneutrality in BAT, whereas it led to an altered reprogramming in response to cold, with enhanced enrichment of different pathways related to metabolism and remodeling. Collectively, we demonstrate a role for Afadin in supporting the adrenergic response in brown adipocytes and BAT function.

## Introduction

Brown adipose tissue (BAT) has a key function as a thermal regulator, due to the distinctive presence of Uncoupling protein (Ucp)-1, which uncouples the electron transport chain to generate heat. BAT is present in adult humans^1–3^ and its presence is inversely correlated with BMI^1^. It is an attractive target for the treatment of metabolic disorders due to its unrivalled capacity to uptake and utilize fatty acids and glucose^4^. Indeed, short-term cold exposure has been shown to improve insulin sensitivity in patients with type 2 diabetes^5^. Cold exposure, or mimicking cold with β_3_-agonists^6^, is the primary mode of BAT activation^7^, although meal-induced BAT glucose uptake and thermogenesis has also been reported^8,9^, indicative of insulin as a potent stimulator of BAT thermogenesis. In agreement with this, Heine et al. recently reported that cold-induced lipolysis triggers insulin secretion from the β-cell which is indispensable for efficient energy uptake and thermogenesis in BAT^10^. The molecular interplay between the anabolic effect of insulin and catabolic β_3_-adrenenic signalling in the BAT remains largely unexplored and is generally assumed to be counter-active. Intriguingly, the mammalian target of rapamycin complex (mTORC)-1, a fundamental signalling hub in the insulin signalling pathway^11^, is also activated by β_3_-agonists in an Akt-independent manner in adipocytes, consequently promoting Ucp1 expression^12^, demonstrating that these two pathways crosstalk.

We have recently discovered that the intracellular scaffold protein Afadin, encoded by the *Mllt4* gene, is a negative regulator of insulin signalling in adipose tissues^13^. Insulin-stimulated phosphorylation of Afadin at S1795 allows its docking with the histone deacetylase (HDAC)-6, to promote downregulation of insulin receptor levels, associated with impaired insulin signalling and lipogenesis. In non-adipose tissues, Afadin has been shown to be a critical component of cell–cell contacts in the form of adherens junctions, but also as an important regulator of cell polarization, differentiation, survival, migration and gene transcription^14,15^, demonstrating the pleiotropic functions of this protein.

Since insulin action plays an important role in BAT thermogenesis, we set out to investigate the role of Afadin in the adaptation of BAT to cold exposure. Here we report that Afadin abundance is increased by cold exposure, potentially in a posttranslational manner. Furthermore, we show that Afadin is essential for the β_3_-adrenegic response in brown adipocytes and to maintain body temperature upon acute cold exposure. Afadin depletion in fat tissues led to alterations in the BAT transcriptomic profile at thermoneutrality and following a cold challenge. Thus, our data uncover the role of Afadin in modulating BAT thermogenesis and physiology.

## Results

### Afadin S1795 phosphorylation and protein abundance are increased following cold exposure in brown adipose tissue

**Figure 1:**
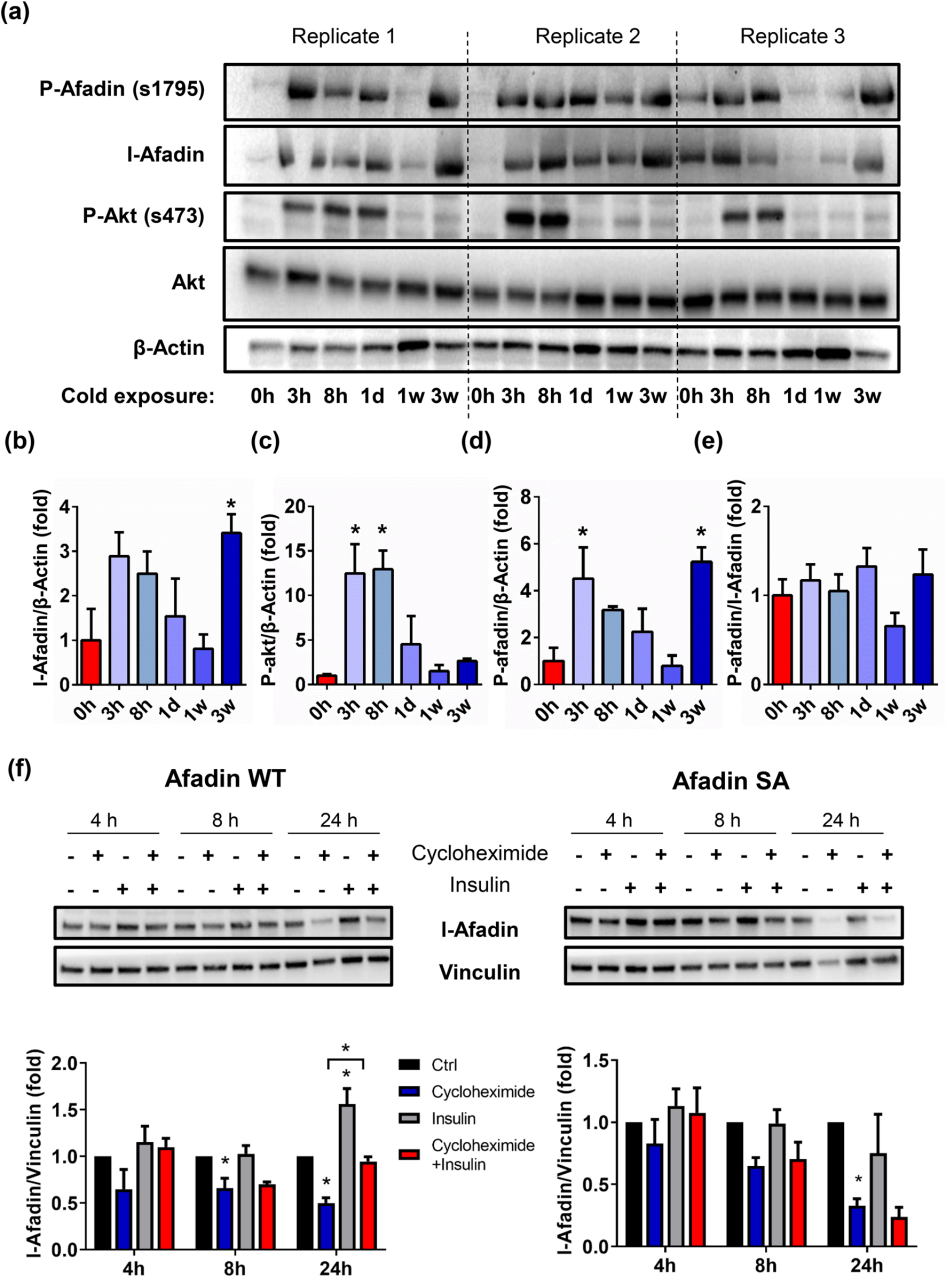
Afadin phosphorylation and abundance are regulated by cold in brown adipose tissue. (**a**) Proteins extracted from BAT were analyzed by SDS-PAGE and (**b**) l-Afadin/β-actin, (**c**) p-Akt//β-actin, (**d**) p-Afadin//β-actin and (**e**) p-Afadin/l-Afadin quantified. (**f**) l-Afadin protein levels in mature adipocytes expressing l-Afadin (Afadin WT) or S1795A l-Afadin (Afadin SA) exposed to vehicle or 10 µg/ml cycloheximide for various time points + /− 100 nM insulin. Representative blots are shown. N = 3. Data are presented as means + SEM, *p < 0.05 vs. 0 h for (**b**–**e**) and versus ctrl or indicated condition in (**f**).

To study the function of Afadin in BAT, we examined its regulation in BAT in response to cold, using mice housed at thermoneutrality for 3 weeks prior to cold exposure for various times^16^. Interestingly, Afadin protein levels displayed a bimodal distribution with an early increase within hours followed by a drop after 1 week of cold and ultimately rising again to a twofold increase after 3 weeks (Fig. 1a,b). Furthermore, the acute cold challenge led to a profound increase in the phosphorylation of Akt at 3 and 8 h (Fig. 1a,c), presumably driven by cold-mediated lipolysis in white adipose tissue stimulating insulin secretion^10^. We have recently shown that Afadin is phosphorylated at S1795 in response to insulin via Akt^13^, and found that Afadin S1795 phosphorylation was also increased upon 3 h of cold exposure (Fig. 1a,d). Notably, the Afadin S1795 phosphorylation followed the same bimodal distribution as the total Afadin protein with an early fourfold rise in phosphorylation after 3 h, a return to basal after 1 week of cold, and a fourfold increase after 3 weeks of cold exposure (Fig. 1a,d). However, normalizing the phosphorylated S1795 Afadin levels to total Afadin levels flattened the change in phosphorylation (Fig. 1e), revealing a strong correlation between the level of S1795 phosphorylation and the abundance of Afadin, and suggesting that Afadin S1795 phosphorylation affects total Afadin protein levels*.* To test this hypothesis, we assessed the turn-over rate of Afadin protein—with or without mutation of S1795 into alanine—in mature adipocytes. Cycloheximide, a protein synthesis blocking agent, led to the gradual decrease of Afadin (~ 50% decrease at 24 h), while insulin led to a slight increase in Afadin protein levels and partially prevented its degradation (Fig. 1f). Interestingly, the insulin-mediated effect on Afadin protein stability was abolished in adipocytes expressing an S1795A mutant, demonstrating that the phosphorylation of S1795 increases Afadin protein content in a posttranslational manner. As opposed to insulin stimulation, stimulation with β_3_-agonists did not lead to increased Afadin S1795 phosphorylation in mature brown adipocytes (Supplementary Fig. 1).

Collectively, these data demonstrate that Afadin protein is regulated by cold exposure in vivo, and suggest that the increase in Afadin protein in cold-exposed mice may be due to enhanced protein stability mediated by insulin via S1795 phosphorylation.

### Afadin regulates β_3_-adrenergic induced gene expression in brown adipocytes

**Figure 2:**
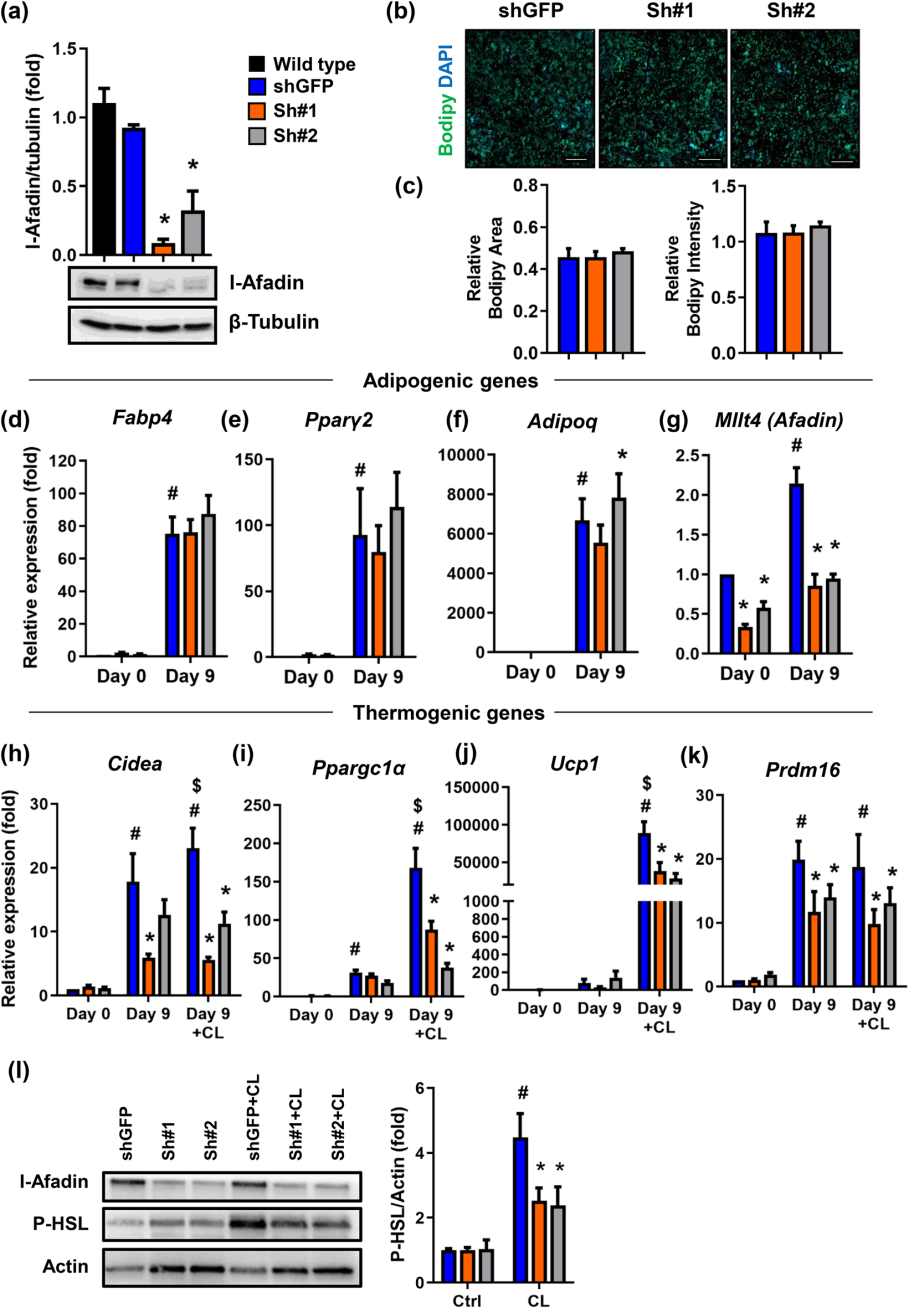
Afadin regulates expression of thermogenic genes in vitro. (**a**) Two stable *Mllt4* shRNA-expressing preadipocyte cell-lines (sh#1 and sh#2) and a GFP shRNA-expressing control preadipocyte cell line (shGFP) were established and knockdown of Afadin confirmed at the protein level. Representative blots are shown. (**b**+**c**) Quantification of Bodipy-staining in differentiated cells. RT-qPCR analysis at day 0 and 9 of differentiation of (**d**) *Fabp4* mRNA, (**e**) *Pparγ2* mRNA, (**f**) *Adipoq* mRNA and (**g**) *Mllt4* mRNA. Mature adipocytes were stimulated with vehicle or 5 μM CL 316,243 (CL) for 4 h. RT-qPCR analysis at day 0 and day 9 of differentiation with or without CL stimulation of (**h**) *Cidea* mRNA, (**i**) *Ppargc1α* mRNA, (**j**) *Ucp1* mRNA and (**k**) *Prdm16* mRNA. (**l**) Immunoblotting from mature adipocytes exposed to vehicle or CL 316,243 for 4 h. N = 3–4. Data are presented as means + SEM, *p < 0.05 vs. shGFP, ^#^p < 0.05 vs. Day 0, ^$^p < 0.05 vs. Day 9.

We next examined the function of Afadin in the brown adipocyte, using shRNA-mediated knockdown of Afadin in preadipocytes. We achieved a 91% (construct sh#1) and 68% (construct sh#2) knockdown efficacy at the protein level compared to the shGFP control cells (Fig. 2a). Next, the cells were differentiated into mature adipocytes and their adipogenic phenotype was evaluated by measuring lipid content (Fig. 2b+c) and the expression of the key adipogenic genes *Fapb4* (Fig. 2d), *Pparγ2* (Fig. 2e) and A*dipoq* (Fig. 2f). In accordance with our previous study^13^, these data show that adipogenesis was not affected by repression of Afadin. Interestingly, there was a twofold increased expression of *Mllt4* –the gene encoding Afadin- upon differentiation (Fig. 2g), further supporting a role for Afadin in the mature brown adipocyte. Since a fundamental feature of the brown adipocyte is the ability to generate heat through mitochondrial uncoupling, we tested the role of Afadin in this process by assessing the expression of key thermogenic genes in Afadin knockdown adipocytes. As expected, the expression of all genes was induced with differentiation, and stimulation with the β_3_-agonist CL 316,243 (CL) led to a further induction of *Cidea*, *Ppargc1α* and *Ucp1* (Fig. 2h–j). Notably, this potentiation was blunted in both cell-lines lacking Afadin, demonstrating that Afadin plays a critical role in the activation of thermogenic genes in the brown adipocyte. In addition, in the absence of β_3_-agonist, Afadin knockdown led to a reduction of *Prdm16* expression (Fig. 2k), indicating that this core transcriptional co-regulator of brown adipocyte development is regulated by Afadin, independently of β_3_-adrenegic stimulation. Consistent with an impaired β_3_-adrenergic response in brown adipocytes lacking Afadin, β_3_-agonist-mediated induction of HSL (Hormone-sensitive lipase) phosphorylation was reduced in Afadin-depleted cells (Fig. 2l).

### Fat-specific loss of Afadin results in impaired cold tolerance

**Figure 3:**
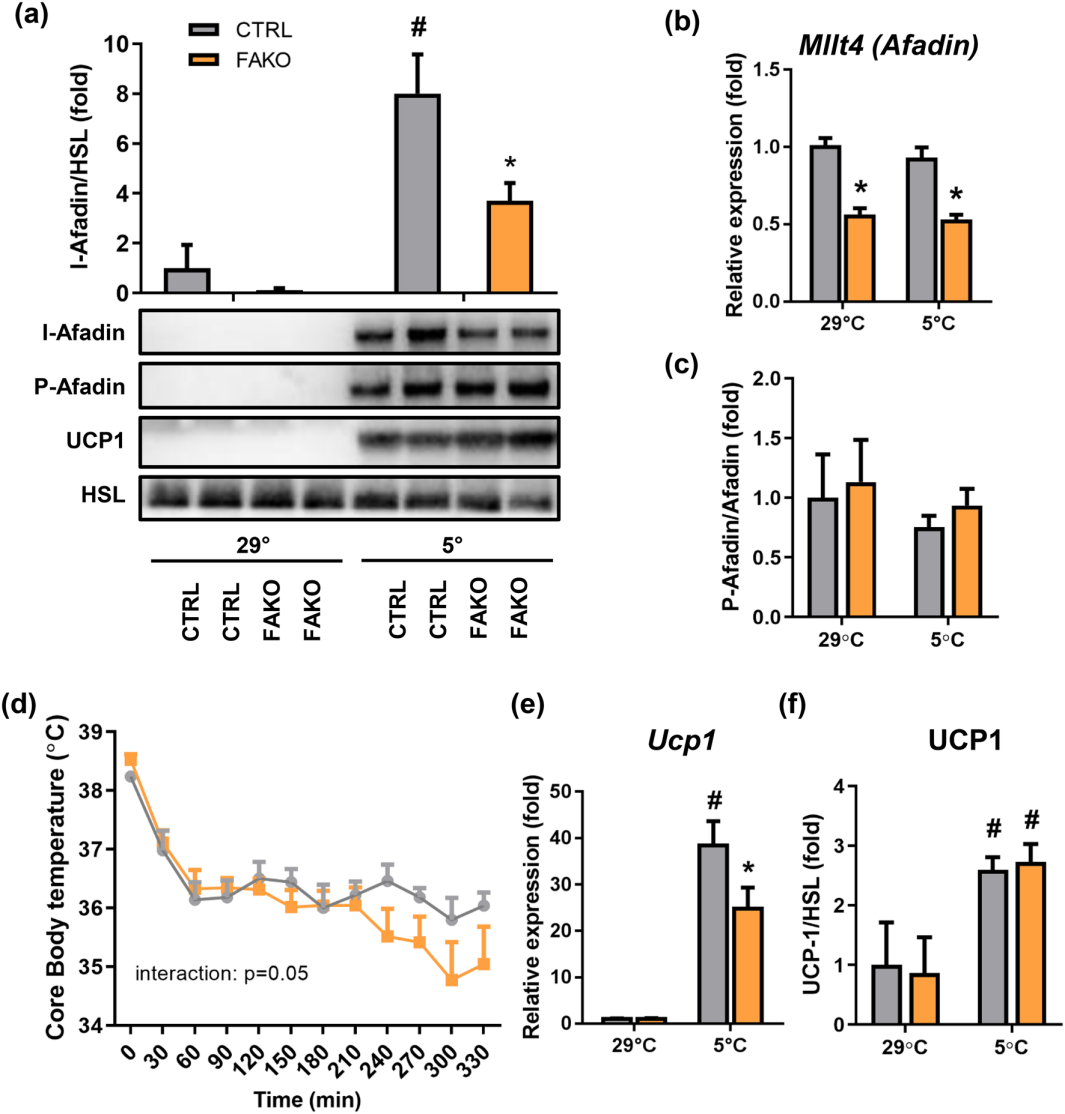
Afadin controls activation of the thermogenic program in vivo. (**a**) l-Afadin, P-Afadin (S1795), Ucp1 and HSL protein levels in fat-specific Afadin knock-out (FAKO) and control littermates (CTRL). Representative blots are shown. (**b**) RT-qPCR analysis of *Mllt4* mRNA levels in FAKO and control littermates. (**c**) Quantification of P-Afadin protein levels. (**d**) Rectal temperature measured every 30 min. (**e**) RT-qPCR analysis of *Ucp1* mRNA levels in FAKO and control littermates. (**f**) Quantification of Ucp1 protein levels. N = 5–7. Data are presented as means + SEM, *p < 0.05 vs. CTRL at 5 °C, #p < 0.05 vs. 29 °C.

Given the Afadin requirement for the regulation of the expression of key thermogenic genes in vitro and the regulation of Afadin by cold exposure in vivo, we next addressed the contribution of Afadin in adaptation to cold. Fat-specific Afadin Knockout (FAKO) mice^13^ and control littermates previously acclimated at thermoneutrality (29 °C) were subjected to an acute cold challenge. As previously reported, Afadin protein levels were increased substantially in BAT from cold-exposed control animals; however this upregulation was reduced in FAKO mice (Fig. 3a, supplementary Fig. 2), indicating that at least part of this regulation occurs in mature adipocytes. Importantly, upregulation of Afadin abundance was not due to an increase in *Mllt4* mRNA (Fig. 3b), demonstrating that Afadin abundance is post-transcriptionally regulated in response to cold. Afadin phosphorylation was also increased in cold-exposed BAT, and paralleled the upregulation of Afadin protein (Fig. 3a+c). As compared to control littermates, FAKO mice had a reduced cold tolerance and, thus, were less capable of defending core body temperature (Fig. 3d). Consistent with our in vitro data, this was paralleled by a defective induction in *Ucp1* expression in cold-exposed BAT from FAKO mice, as compared to control animals (35% decrease, Fig. 3e). However, this defect was not observed at the protein level (Fig. 3a+f). Together, these data show that Afadin is necessary for cold-induced thermogenesis, via Ucp1 protein independent mechanisms.

### Transcriptional profiling of Afadin-ablated BAT reveals changes in regulation of metabolic pathways at thermoneutrality and in response to cold

**Figure 4:**
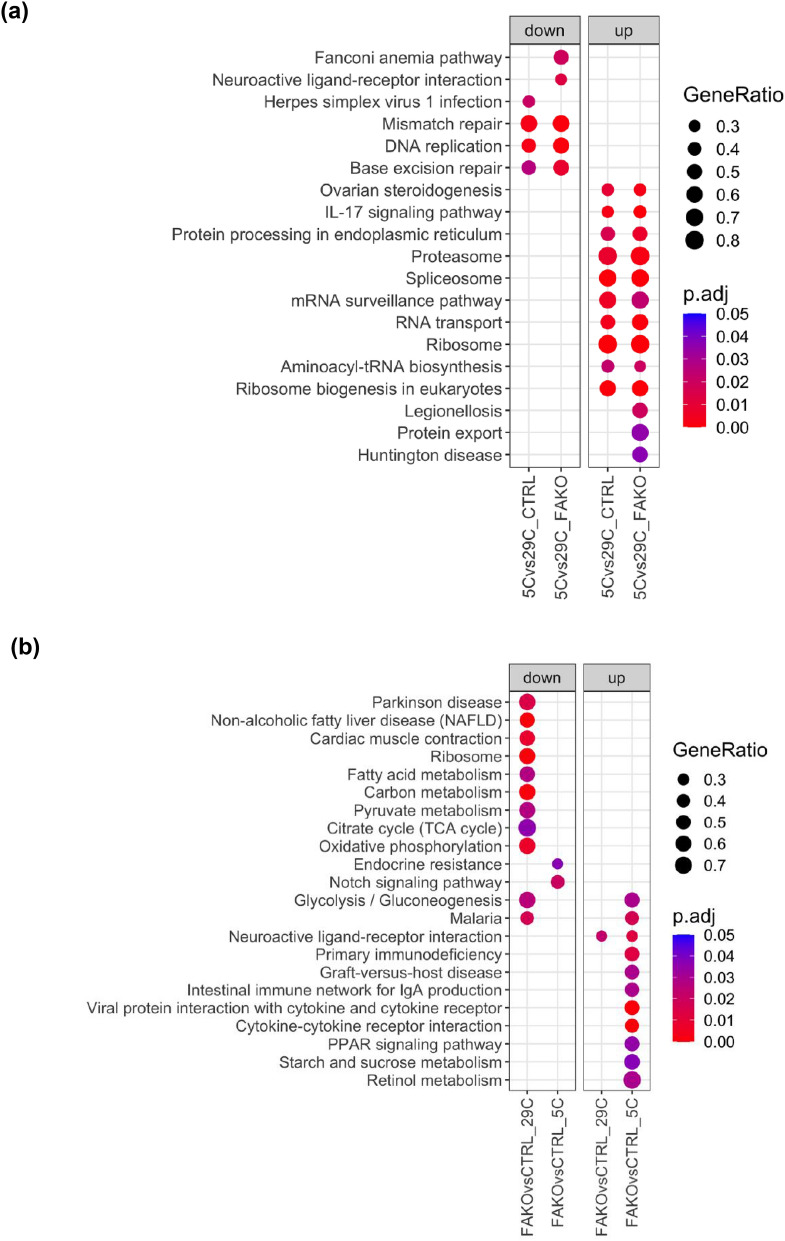
Afadin deletion leads to transcriptomic alterations in BAT at thermoneutrality and in response to cold. Male fat-specific Afadin knock-out (FAKO) and control littermates (CTRL) were housed at thermoneutrality (29 °C) for 2 weeks followed by 5 °C cold-exposure for 6 h and BAT was harvested and RNA-sequencing was performed. (**a**,**b**) Gene set enrichment analysis (GSEA) for each comparison. Top 10 significant up/down regulated KEGG terms containing at least 10 genes were plotted using gene ratio, regulated by temperature (**a**) and in between genotypes (**b**). Full list of enriched KEGG terms can be found in Supplementary Table 1.

**Figure 5:**
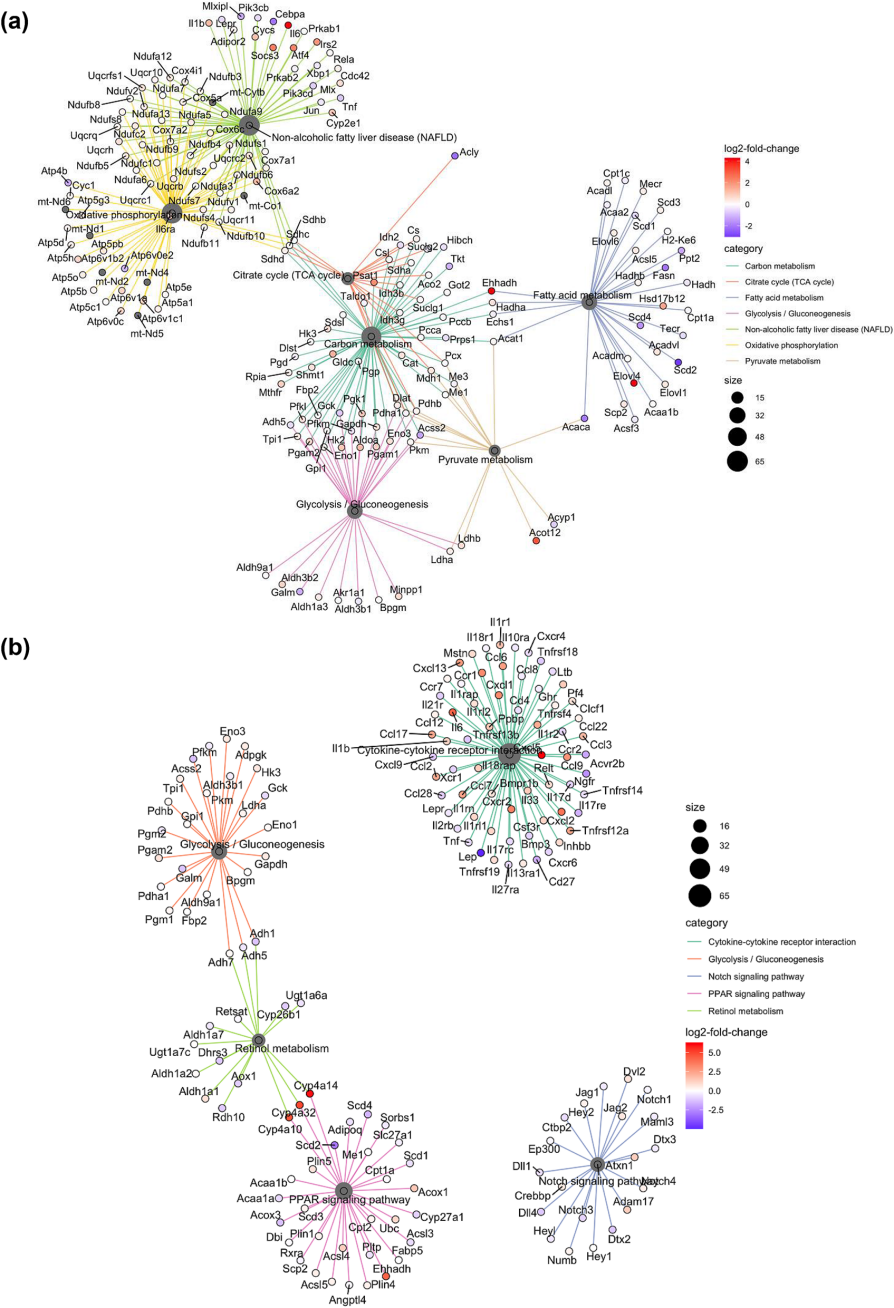
Afadin deletion modulates the transcriptomic profile within specific pathways regulating BAT function. (**a**,**b**) KEGG term—gene interaction plot depicting the linkages of genes and biological concepts (KEGG terms) as a network. Log2 fold changes (FAKO vs CTRL) of gene expression within selected enrichments at 29 °C (**a**) or at 5 °C (**b**).

To uncover the mechanisms underlying BAT dysfunction in FAKO mice, we compared the transcriptomic response of BAT from control and FAKO mice transitioning from thermoneutrality to a cold environment. BAT transcriptional profile was dramatically reprogrammed following cold challenge, independently of the genotype (Fig. 4a, Supplementary Fig. 3, Supplementary Table 1). Further, gene set enrichment analysis (GSEA) revealed significant up/down regulated KEGG terms between control and FAKO mice (Fig. 4b). The most striking observation when comparing the annotated pathways at thermoneutrality between Afadin-depleted BAT and control BAT, was the general downregulation in pathways associated with metabolism such as “Glycolysis/Gluconeogenesis”, “Fatty acid metabolism”, “Carbon metabolism”, “Pyruvate metabolism”, “Non-alcoholic fatty liver disease (NAFLD)” and “Oxidative phosphorylation” (Fig. 5a). Yet, in response to cold, few pathways such as “Endocrine resistance” and “Notch signalling pathway” were downregulated in FAKO animals as compared to controls, whereas several pathways, including “Glycolysis/Gluconeogenesis”, “Retinol metabolism”, “PPAR signalling pathway” and “Cytokine-cytokine receptor interaction” were upregulated (Figs. 4b, 5b). Thus, in the absence of Afadin, the BAT transcriptomic profile was altered at thermoneutrality and inappropriately remodeled in response to cold, with opposite expression patterns of pathways supporting important metabolic and signaling functions.

## Discussion

Non-shivering thermogenesis is a key feature of BAT allowing heat production at the expense of lipid and glucose utilization^4^. In the present study, we show that the abundance and phosphorylation of the scaffold protein Afadin is induced by cold in BAT, and it is required for adequate adaptation to acute cold exposure.

Afadin was originally described as an adherens junction-associated protein^17^, however we have recently shown that Afadin functions as a negative regulator of insulin signalling in adipocytes and adipose tissue via insulin-mediated phosphorylation of Afadin at S1795^13^. Here, we show that Afadin is also regulated by cold exposure via phosphorylation, suggesting an important role for Afadin in cold adaptation. Increased phosphorylation of Afadin at S1795 may occur in response to activation of the insulin signalling pathway via cold-induced lipolysis^10^, through Akt, an essential component of the BAT response to cold^18^; or via S6 kinase 1, downstream of mTORC1, as recently demonstrated^19^. Notably, we demonstrate that the rapid induction in Afadin protein happens in a posttranscriptional manner, and may be due to increased protein stability, as suggested by reduced stability of a non-phosphorylatable Afadin mutant (S1795A) in brown adipocytes, and in accordance with previous studies indicating that S1795 phosphorylation increases Afadin half-life^20^. However, insulin- and refeeding-stimulated phosphorylation of Afadin in adipose depots were not accompanied with increased protein abundance, suggesting that additional mechanisms induced upon cold exposure, possibly acting in conjunction with phosphorylation at S1795, are necessary to increase protein abundance, and warrant further investigation. Lastly, the possibility that cold exposure results in increased abundance of Afadin via independent mechanisms, allowing Afadin to be more abundantly phosphorylated, cannot be excluded. Also, BAT is a highly plastic organ displaying pronounced cellular remodeling and structural changes upon temperature changes^21^, which may be responsible for the increased Afadin content in BAT after prolonged cold exposure. Both Afadin abundance and insulin-induced phosphorylation at S1795 were reduced in BAT from obese mice^13^. Thus, it would be interesting to study whether obesity similarly interferes with the cold-regulation of Afadin.

In turn, we found that Afadin is involved in the thermogenic response in brown adipocytes. Indeed, Afadin repression impaired the beta adrenergic response and reduced induction of thermogenic genes including *Ucp1 *in vitro, and weakened the cold-induction of *Ucp1* mRNA in vivo. Thus, Afadin modulates β_3_-activation in a cell-autonomous manner in the mature brown adipocyte. Despite clear effects on *Ucp1* mRNA, Ucp1 protein was not affected by Afadin ablation, suggesting that the inability to maintain body temperature during cold exposure, at least at this time point, is not due to defective Ucp1 upregulation. On the other hand, our transcriptional analysis revealed significant dysregulation of several metabolic pathways including *glycolysis/gluconeogenesis* and *oxidative phosphorylation* in BAT from FAKO mice at thermoneutrality. Collectively, these alterations are indicative of a defective BAT transcriptomic profile^22^, possibly preventing an adequate response of the tissue when the animals are challenged by cold and resulting in insufficient thermogenic response and inability to maintain normal body temperature. In addition, the transcriptional reprogramming occurring in BAT in response to cold was altered with Afadin ablation. In particular, dysregulation of *PPAR signalling pathway*, *glycolysis/gluconeogenesis* and *starch and sucrose metabolism* suggest an impaired metabolic response in BAT when Afadin fails to be upregulated. Moreover, downregulation of *Notch signaling pathway*, and upregulation of inflammation-related pathways suggest a tissue functional impairment or a compensational mechanism to cope with an inappropriate response. While the detailed molecular mechanisms underlying Afadin regulation of transcriptional regulation of metabolic pathways remain to be determined, our results suggest that a reduced cellular response to β_3_-adrenergic stimulation contributes to impaired regulation of metabolic genes, including *Ucp1*. In addition, altered expression of key transcriptional regulators, such as Prdm16, may impede the priming of brown adipocytes, resulting in an inappropriate transcriptional response.

Importantly, BAT activation and adaptation to cold is a complex process where lipogenesis and lipid oxidation are concurring, at least in part via insulin signalling and PPAR signalling^18^, further highlighting the need for balancing both anabolic and catabolic activities. Further studies are needed to clarify the potential contribution of Afadin in adequately harmonizing these events. In addition, posttranscriptional defects not investigated in the current study may be responsible for BAT dysfunction in FAKO mice. Finally, since Afadin was also lacking in white adipose tissue (WAT) in our animal model, and that we previously reported a role for Afadin in the regulation of adipose insulin action^13^, a participation of WAT dysfunction in the dysregulation of whole-body energy balance cannot be ruled out.

Collectively, the present data identifies Afadin as an important regulator of BAT adaption to acute cold. In combination with our recent observation of Afadin being a negative regulator of insulin action^13^, this suggest that, in the brown adipocyte, Afadin functions as a nexus controlling both anabolic (insulin signalling) and catabolic (β_3_-signalling) responses to physiological stimuli.

## Materials and methods

### Reagents

Antibodies: Phospho-Afadin (#5485), Total β-Actin (#8457), Total β-Tubulin (#2128), Phospho-Akt (##9271) and Total Akt (##9272) were from Cell Signaling. Total l/s-Afadin (A0224) was from Sigma. Chemicals: Insulin, T3, IBMX, Dexamethasone, Indomethacin, puromycin, cycloheximide and CL 316,243 were from Sigma. DAPI was from Cell Signaling, and BODIPY 493/503 was from ThermoFisher. Oligonucleotides: Mouse primers (TAG Copenhagen and IDT) are listed in Table 1. MLLT4 MISSION shRNA TRCN0000090486 and TRCN0000090487 were from Sigma. shGFP construct was from GE Dharmacon.

**Table 1:** Primers for RT-qPCR.

Gene	Forward primer	Reverse primer
*Adipoq*	GGCAGGAAAGGAGAACCTGG	AGCCTTGTCCCTCTTGAAGAG
*Arbp*	TTTGGGCATCACCACGAAAA	GGACACCCTCCAGAAAGCGA
Cidea	ATCACAACTGGCCTGGTTACG	TACTACCCGGTGTCCATTTCT
*Fabp4*	GATGCCTTTGTGGGAACCT	CTGTCGTCTGCGGTGATTT
*Mllt4*	GGCCTTCTCAAGGGGATGAC	AAAGCTGGTCTCAGGCATGT
*Pparγ2*	TCAGCTCTGTGGACCTCTCC	ACCCTTGCATCCTTCACAAG
*Ppargc1α*	CCCTGCCATTGTTAAGACC	TGCTGCTGTTCCTGTTTTC
*Prdm16*	CAGCACGGTGAAGCCATTC	GCGTGCATCCGCTTGTG
*Ucp1*	CTGCCAGGACAGTACCCAAG	TCAGCTGTTCAAAGCACACA
*18 s*	AGTCCCTGCCCTTTGTACACA	GATCCGAGGGCCTCACTAAAC

### Animal models

All mice were on a C57BL/6 J background. Adiponectin-Cre mice were from the Jackson Laboratory (The Jackson Laboratory, Bar Harbor, ME; USA) and Mllt4^flox/flox^ mice were provided by Pr. Müller^23^. Mice were housed at the Department of Experimental Medicine, Faculty of Health Sciences, at the University of Copenhagen at 22 °C under daily 12 h light/dark cycles in ventilated racks, with free access to food and water, and cages changed every 2 weeks^13^. For cold exposure experiments, 12–16 weeks old mice were individually housed and placed into climate controlled rodent incubators (Memmert HPP750Life) at 29 °C to acclimate to thermoneutrality for 14–18 days. Cold-exposed mice were then moved to an incubator set to 5 °C with food and water ad libitum, until termination and dissection. All animal experiments were approved by the Danish Animal Experiments Inspectorate (permit number 2015-15-0201-00728), and performed in accordance with the guidelines and regulations of the Department of Experimental Medicine at the University of Copenhagen.

### Cell culture

The brown preadipocyte cell-line established by Pan et al.^24^ was a kind gift from Dr. Yong-Xu Wang. Cells were maintained in DMEM high glucose with 10% fetal bovine serum, 1% Penicillin and Streptomycin (complete media). DE cells were differentiated using the published protocol^25^ with the exception of 2% FBS; briefly, cells were grown to confluence in 20 nM insulin and 1 nM T3. Two days after confluence, cells were induced by adding 20 nM insulin, 1 nM T3, 0.5 mM IMBX, 0.5 μM Dexamethasone and 125 mM Indomethacin for 2 days. Cells were then switched back to 20 nM insulin and 1 nM T3 for 4 days with media change every other day. Cells were considered mature 8 days after induction and kept in complete media for 1 day before experiments. Mature adipocytes were stimulated with 5 μM CL-316,243 or vehicle for 4 h. For cycloheximide experiments, Afadin KO cells with re-expression of l-Afadin or S1795A Afadin^13^ were used. For Norepinephrine/Isoproterenol stimulation, WT-1 brown adipocytes^25^ (a kind gift from Dr. C. Ronald Kahn) were used. Mature adipocytes were used at day 9 and kept in complete media with or without insulin stimulation throughout the study period.

### Lentiviral-mediated knockdown

Stable shRNA-expressing cell-lines were established as previously described^26^. Infected cells were selected using Puromycin (2.5 μg/ml) for seven days. Selection pressure was kept using 1 μg/ml while passaging cells, but not during experimental setups.

### Immunoblotting

Immunoblotting was performed as previously described^13^. In brief, cells or tissues were lysed, proteins collected and denatured. Equal amounts of proteins were resolved by SDS-PAGE and transferred to PVDF membranes. Membranes were blocked in 5% BSA for one hour followed by incubation overnight with primary antibody in a TBST solution with 1% BSA.

### RNA extraction and qPCR analysis

RNA from cultured cells was extracted as previously described^13^, using the RNeasy kit (74106, Qiagen) and cDNA synthesized using the High Capacity cDNA Reverse Transcription kit (ThermoFisher) according to the manufacturer’s instructions. RNA from tissue was extracted using steel bead homogenization in Trizol (Invitrogen), as previously described^13^. Briefly, the aqueous phase was retrieved after chloroform extraction and mixed 1:1 with 70% ethanol. The samples were then transferred to RNeasy columns, RNA was purified according to the manufacturer’s instructions—including DNase treatment-, and cDNA was synthesized using the iScript kit (BIO-RAD). Real-time PCR was performed as previously described^13^, using either the 7900HT Real-Time PCR System (ThermoFisher) or the CFX384 Real-Time System according to the supplier’s manual (BIO-RAD). Each cDNA sample was analyzed in triplicates. Target gene expression was normalized to *Arbp* (cell-line experiments) or *18 s* (mouse experiments) mRNA. Primer sequences are described in Table 1.

### Bodipy staining

DE cells were plated and differentiated in 24-well-plates and were stained with BODIPY 493/503 and DAPI to assess lipid accumulation/content. Cells were fixated in 4% paraformaldehyde for 15 min at room temperature, washed twice in PBS and then stained with 10 μg/ml Bodipy 493/503 (lipid droplets) and 1 μg/ml DAPI (nuclear staining) for 15 min. Lastly, cells were washed and imaged with Zeiss Cell Observer microscope with a 10X objective. 9 randomly selected fields of view per well were imaged and all images were acquired with exact same settings. Analysis of the bodipy area and intensity and cells surface area was performed in ZEN software. The relative area/intensity was calculated by dividing the Bodipy area or intensity for the area occupied by the cells (cells surface area) in each field of view.

### RNA sequencing

Total RNA (600 ng) was depleted from rRNA by applying the Ribo-Zero Magnetic Gold Kit (Epicentre, WI, USA). The Agencourt RNAClean XP (Beckman Coulter, CA, USA) was used to purify the rRNA-depleted samples. Libraries were then prepared as described previously^27^, with the TruSeq Stranded Total Sample Prep Kit (Illumina, CA, USA). The quality of the libraries were controlled with a Bioanalyzer instrument (Agilent Technologies) and then subjected to 100-bp single-end sequencing on a NextSeq 500 system (Illumina). An average depth of 30 million reads per sample was obtained.

### Bioinformatic analysis

RNA-seq raw reads were aligned to mouse genome (mm10) using STAR v2.5.2b resulting ~ 23 M aligned reads on average. Gene coverages were calculated by featureCounts v1.5.2^28^ using GENCODE^29^ annotation release M13 with an average of 17 M assigned reads. Genes with low expression were removed for differential expression analysis with filterByExpr which is a built-in edgeR function. Differentially expressed genes (DEGs) were computed using edgeR v3.22.0^30^ with the glmQLFit /glmQLFTest function using a model of the form *y ∼* *0* + *group*, where *group* represents the unique combinations of genotype and temperature of mice adipose tissue sample. Genes having a false discovery rate (FDR) below 0.05 were considered differentially expressed. Gene set enrichment analysis (GSEA) was performed using clusterProfiler v3.10.0^31^ with KEGG database using the same FDR threshold (FDR < 0.05)^32^. As several KEGG terms were significant across different contrasts, we have selected top 10 significant KEGG terms (sorted by gene ratio, which is defined as the number of present genes in our dataset divided by the number of genes registered in KEGG database for the given KEGG term) for both up and down-regulated terms while considering at least 10 genes should be present in our gene expression dataset. The RNA-seq data is publicly available on GEO database with the accession number: GSE147392.

### Statistics

All experiments use biological replicates, where N indicates the number of animals used, or the number of times the experiment was repeated. Data are presented as means + SEM and statistical tests were performed using the software GraphPad Prism. For comparisons between two groups, Student’s t-test was used, whereas comparisons between more than two groups statistical analysis was carried out by ANOVA followed by post-hoc multiple correction tests. Results were considered significant for P-values below 0.05.

## Supplementary Information

Supplementary Information 1.

Supplementary Information 2.

## Data Availability

The RNA-sequencing data are publicly available on GEO database with the accession number: GSE147392. The data that support the findings of this study are available from the corresponding author upon reasonable request.
