# Supplementary material for: Cold-induction of afadin in brown fat supports its thermogenic capacity
Source: Sci Rep. 2021 May 7;11:9794. doi: 10.1038/s41598-021-89207-2 (PMC8105362; doi:10.1038/s41598-021-89207-2)

Cold-Induction Of Afadin In Brown Fat Supports Its Thermogenic Capacity

Morten Lundh[1,2], Ali Altıntaş[1§], Marco Tozzi[1§], Odile Fabre[1], Tao Ma[1], Farnaz Shamsi[2], Zachary Gerhart-Hines[1], Romain Barrès[1], Yu-Hua Tseng[2], Brice Emanuelli[1*]

[1] Novo Nordisk Foundation Center for Basic Metabolic Research, Faculty of Health and Medical Sciences, University of Copenhagen, Copenhagen, Denmark.

[2] Joslin Diabetes Center, Harvard Medical School, Boston, Massachusetts, USA.

[§]These authors contributed equally

[*]Correspondence and lead contact:

Brice Emanuelli, e-mail: emanuelli@sund.ku.dk

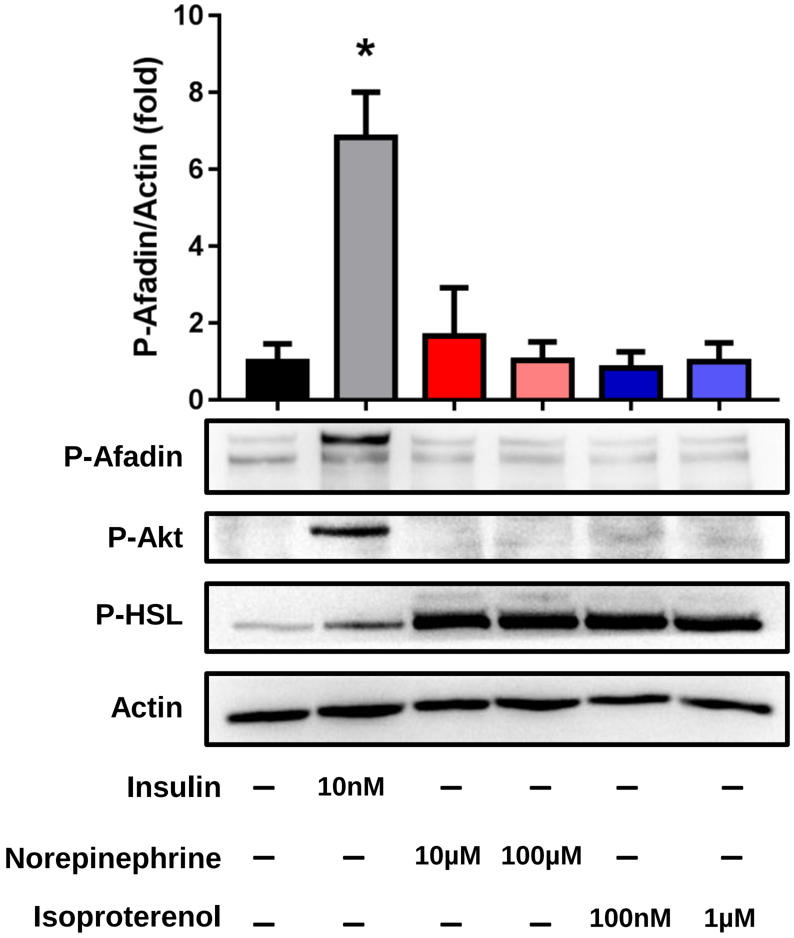

Supplementary Figure 1: Norepinephrine and Isoproterenol stimulation do not induce S1795 phosphorylation of Afadin. P-Afadin protein levels in mature adipocytes exposed to vehicle, insulin, norepinephrine or isoproterenol for 15 minutes. Representative blots are shown. N=4. Data are presented as means + SEM, *$p<0.05$

Supplementary Figure 1

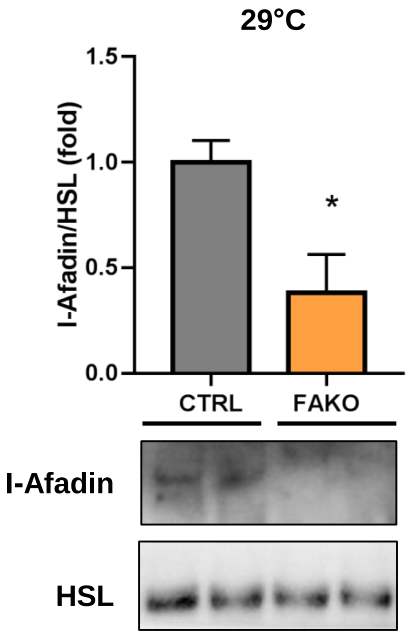

Supplementary Figure 2: BAT Afadin abundance at thermoneutrality. l-Afadin and HSL protein levels in fat-specific Afadin knock-out (FAKO) and control littermates (CTRL). Representative blots are shown. N=6. Data are presented as means + SEM, *p<0.05

Supplementary Figure 2

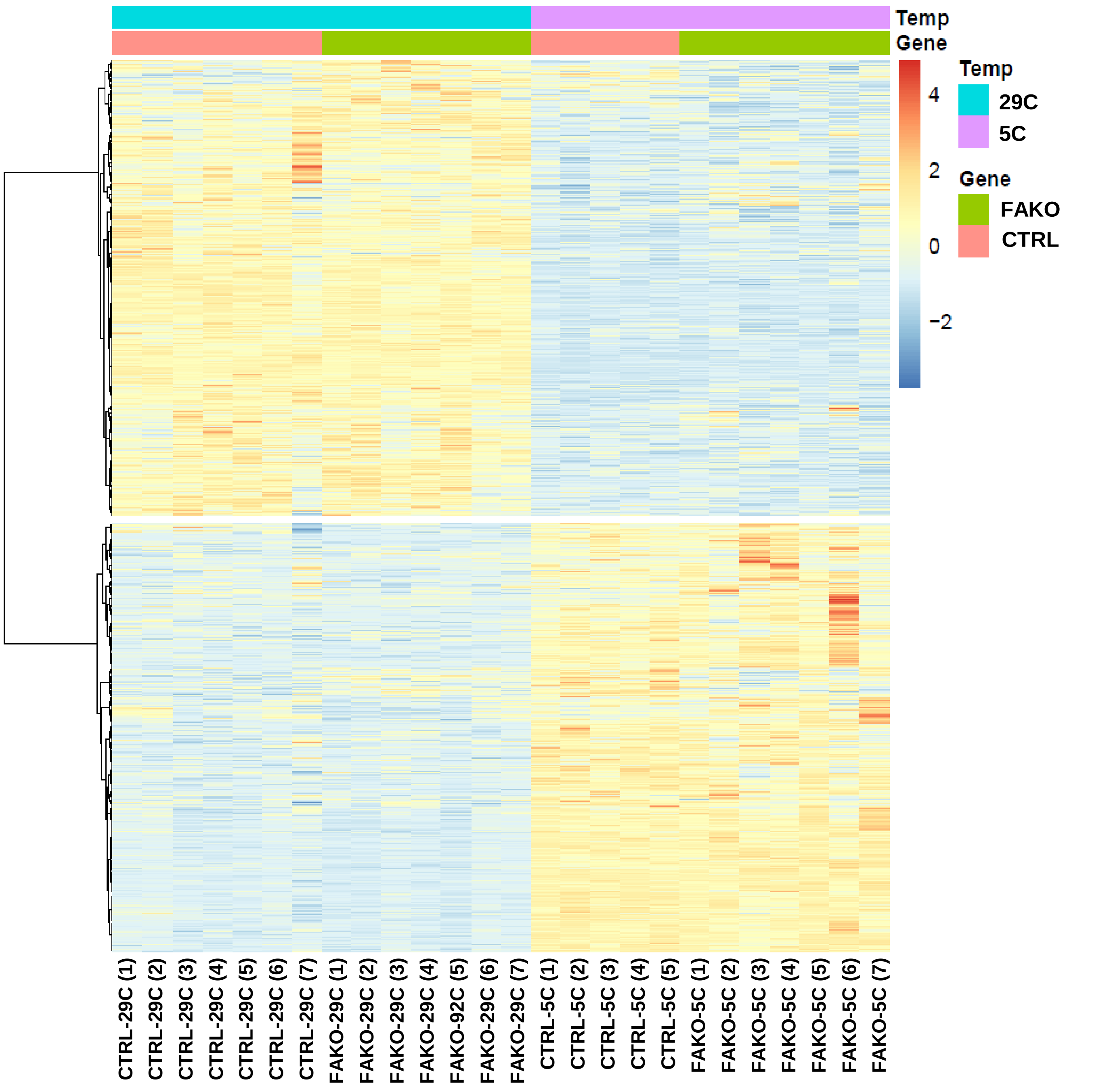

Supplementary Figure 3: Afadin deletion modulates the transcriptomic profile in BAT at thermoneutrality and in response to cold. Male fat-specific Afadin knock-out (FAKO) and control littermates (CTRL) were housed at thermoneutrality (29°C) for 2 weeks followed by 5°C cold-exposure for 6 hours and BAT was harvested and RNA-sequencing was performed. Heatmap showing hierarchical clustering of all differentially expressed genes. Z-scores are calculated for each gene. Hierarchical clustering was performed using "ward.D2" method using Pearson distance.

Supplementary Figure 3

Supplementary information

full-length blots used to generate the figures are provided here

Figure 1 (a)

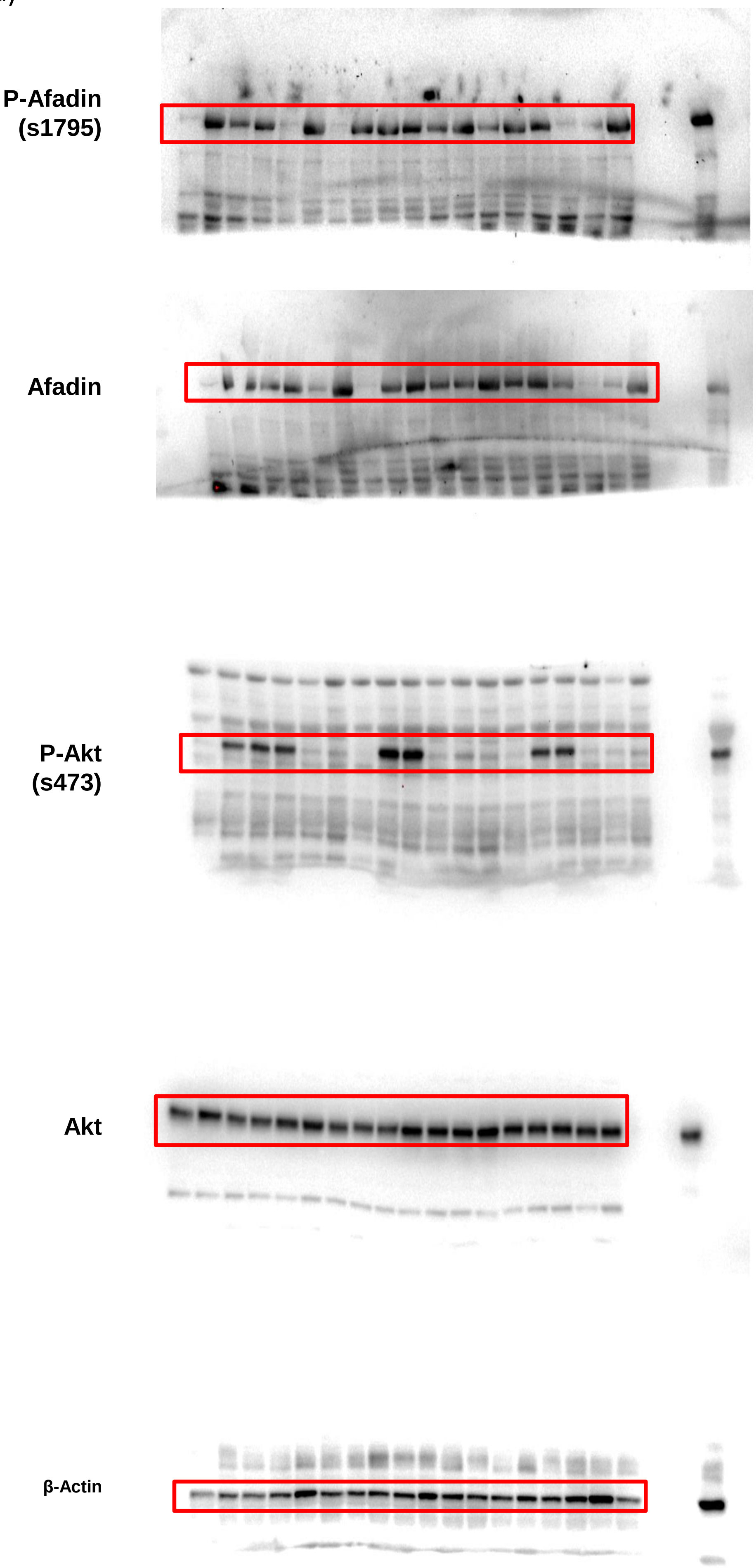

Figure 1(f)

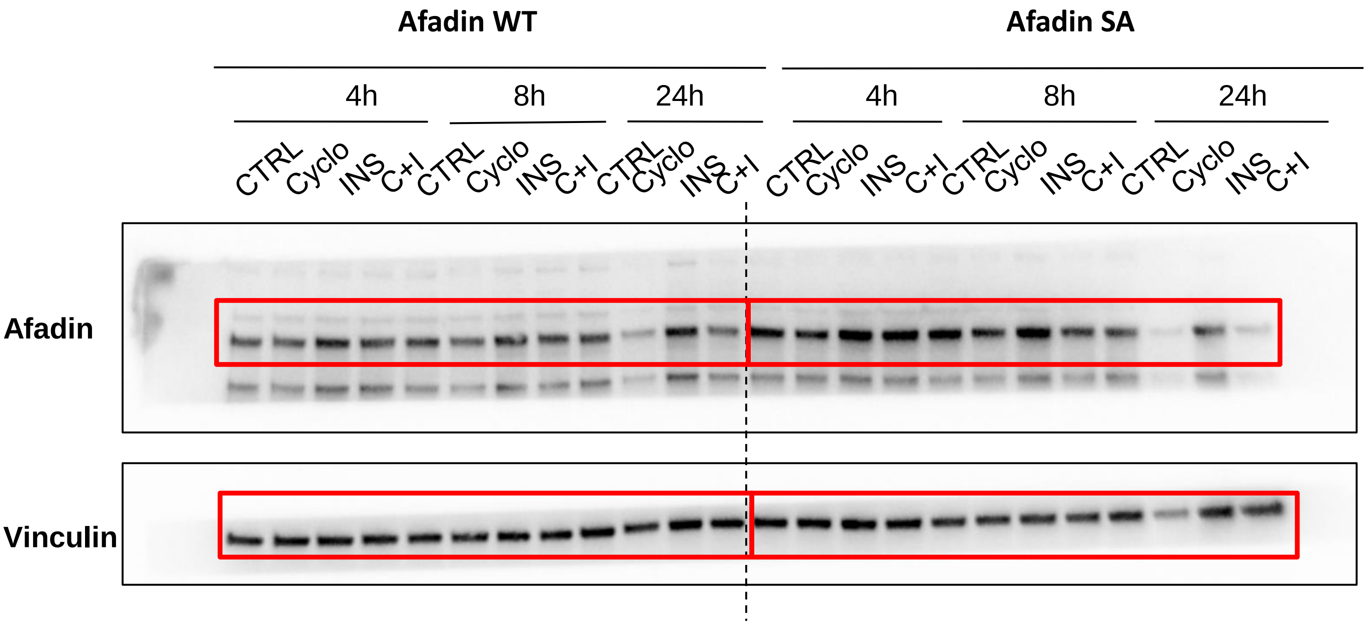
Afadin WT
Afadin SA
4h
8h
24h
4h
8h
24h
CTRL Cyclo INS C+I CTRL Cyclo INS C+I CTRL Cyclo INS C+I
CTRL Cyclo INS C+I CTRL Cyclo INS C+I CTRL Cyclo INS C+I
Afadin
Vinculin

Figure 2(a)

Afadin

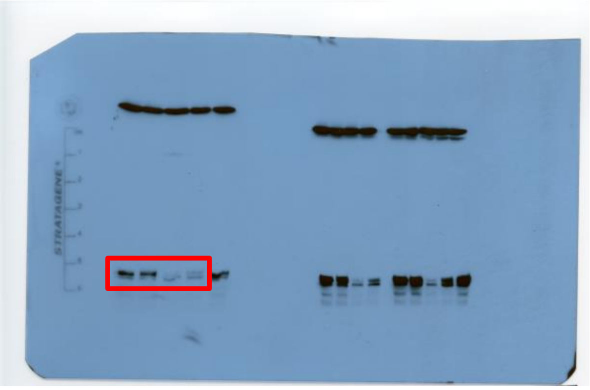

β-tubulin

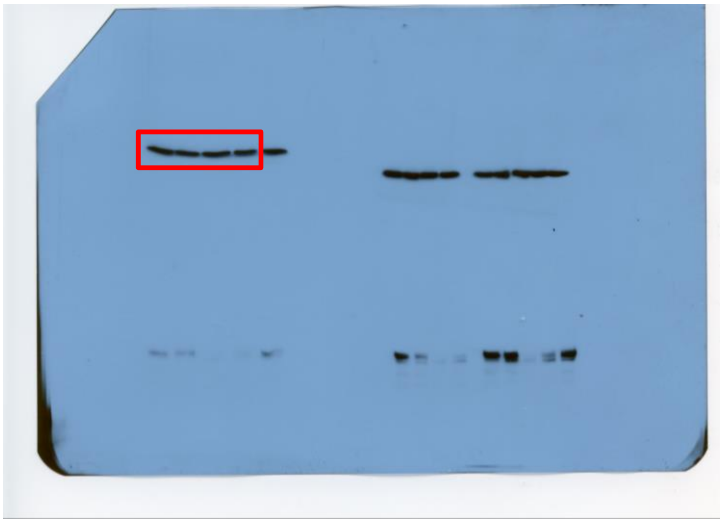

Figure 2(l)

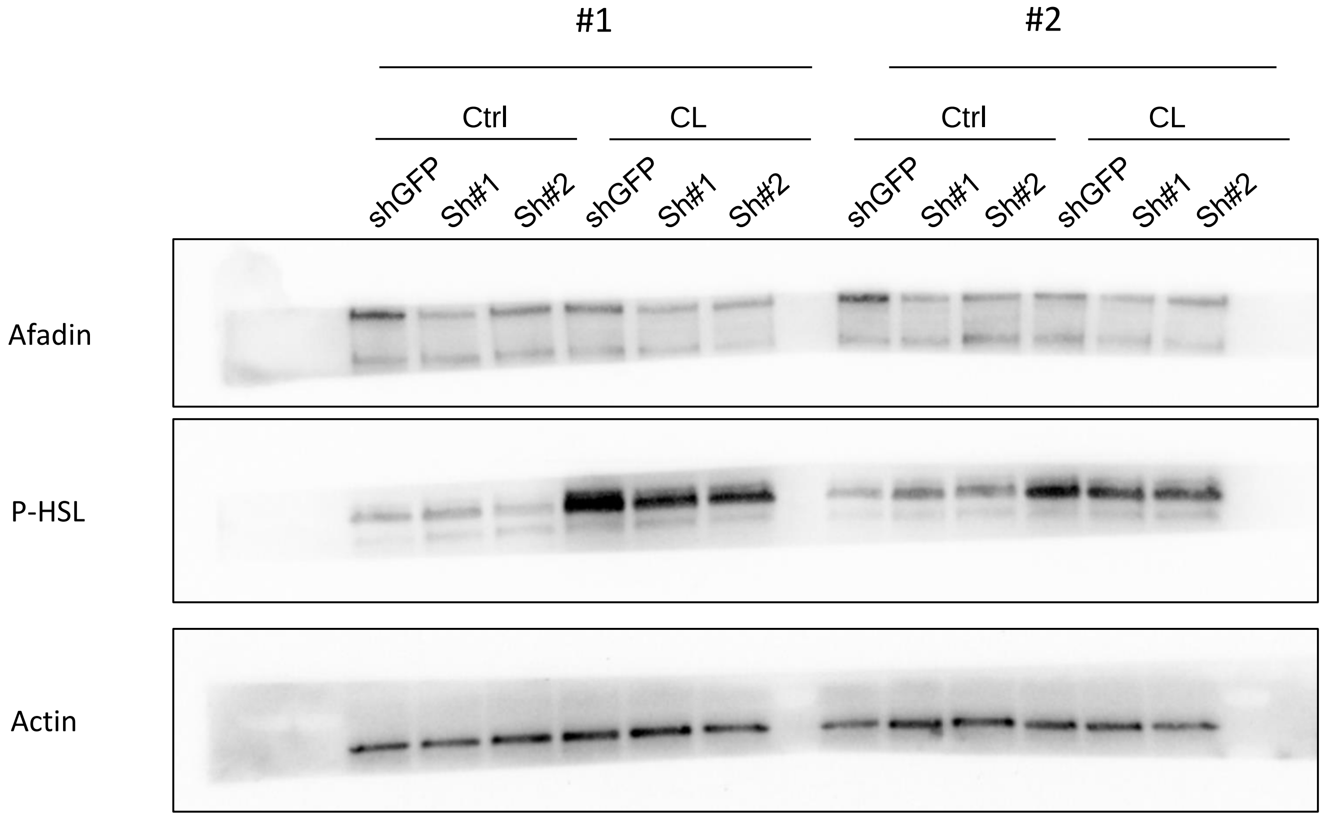

#1
#2
Ctrl
CL
Ctrl
CL
shGFP
Sh#1
Sh#2
shGFP
Sh#1
Sh#2
shGFP
Sh#1
Sh#2
shGFP
Sh#1
Sh#2
Afadin
P-HSL
Actin

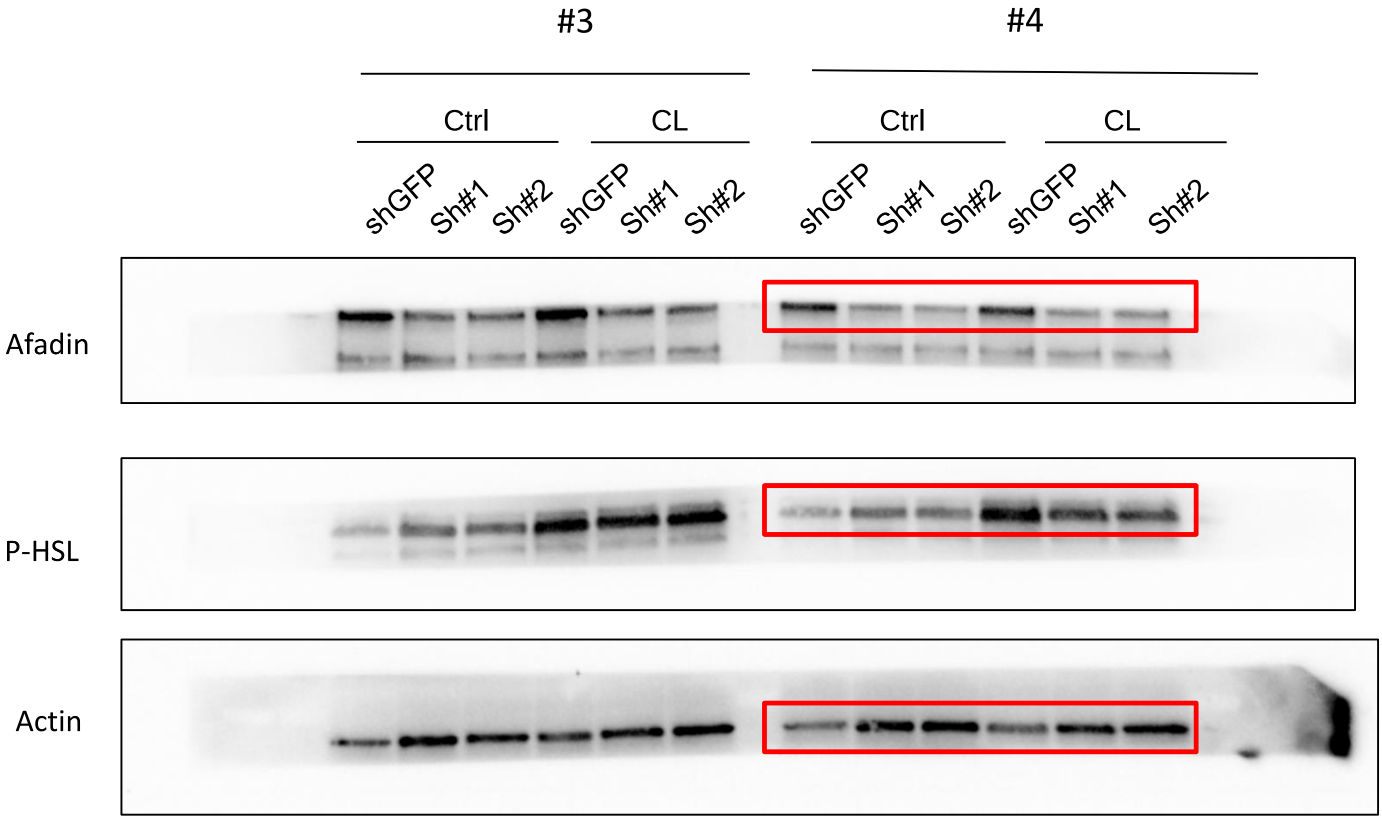

#3
#4
Ctrl
CL
Ctrl
CL
shGFP
Sh#1
Sh#2
shGFP
Sh#1
Sh#2
shGFP
Sh#1
Sh#2
shGFP
Sh#1
Sh#2
Afadin
P-HSL
Actin

Figure 3(a)

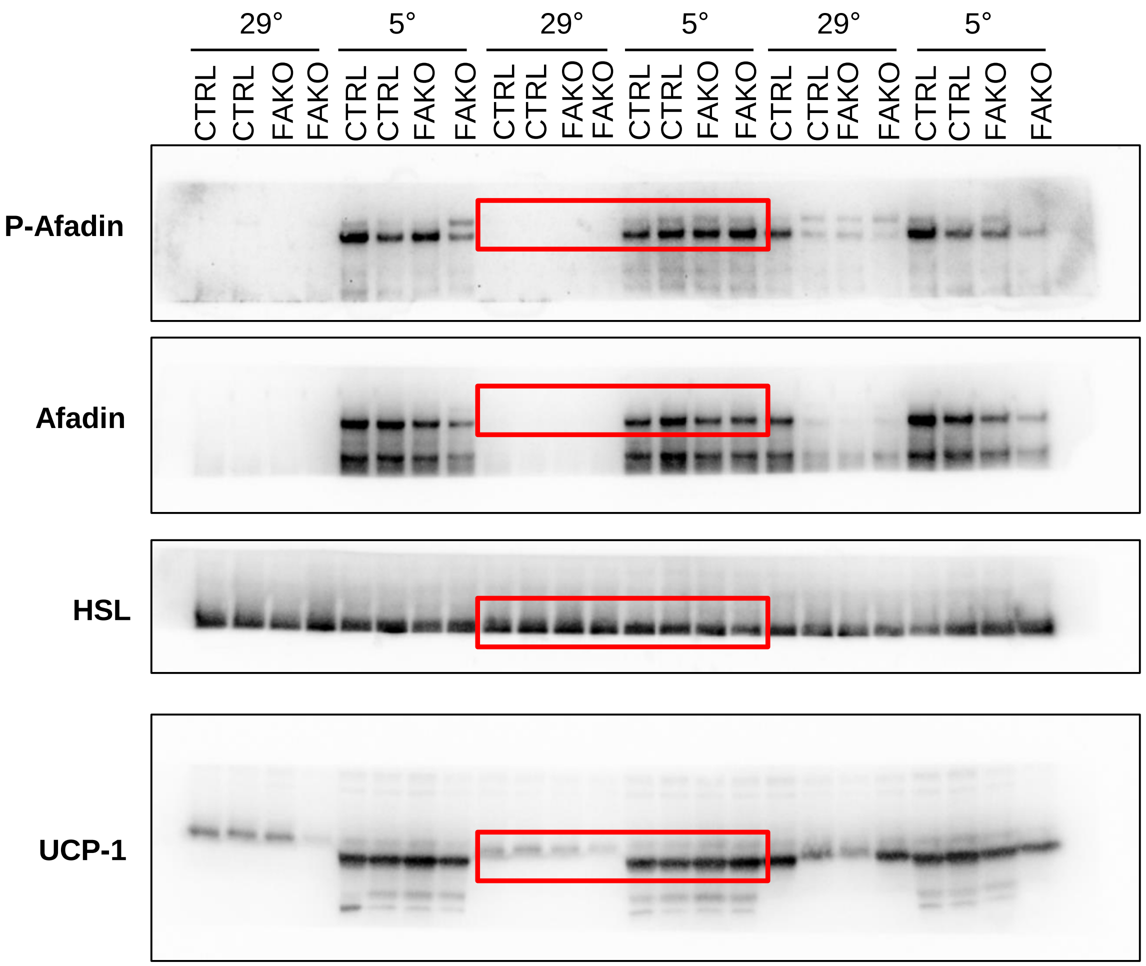
29°
5°
29°
5°
29°
5°
CTRL CTRL FAKO FAKO CTRL CTRL FAKO FAKO CTRL CTRL FAKO FAKO CTRL CTRL FAKO FAKO CTRL CTRL FAKO FAKO CTRL CTRL FAKO FAKO
P-Afadin
Afadin
HSL
UCP-1

Supplementary Figure 1

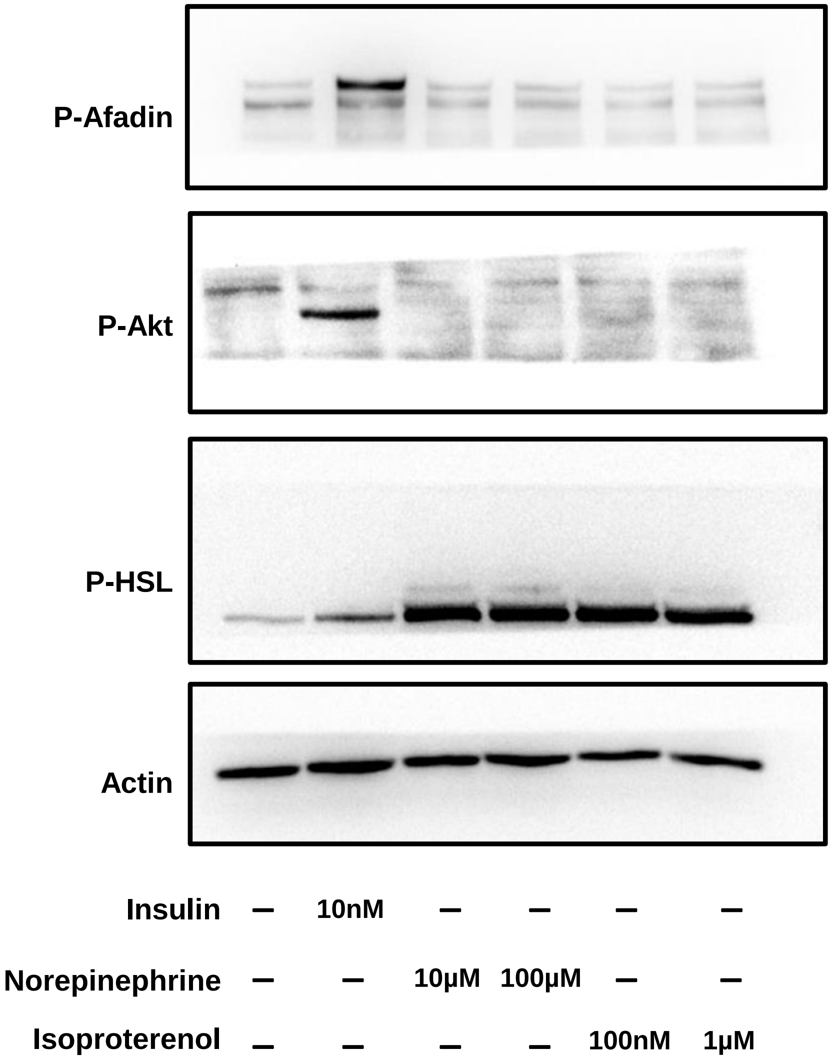

Supplementary Figure 2

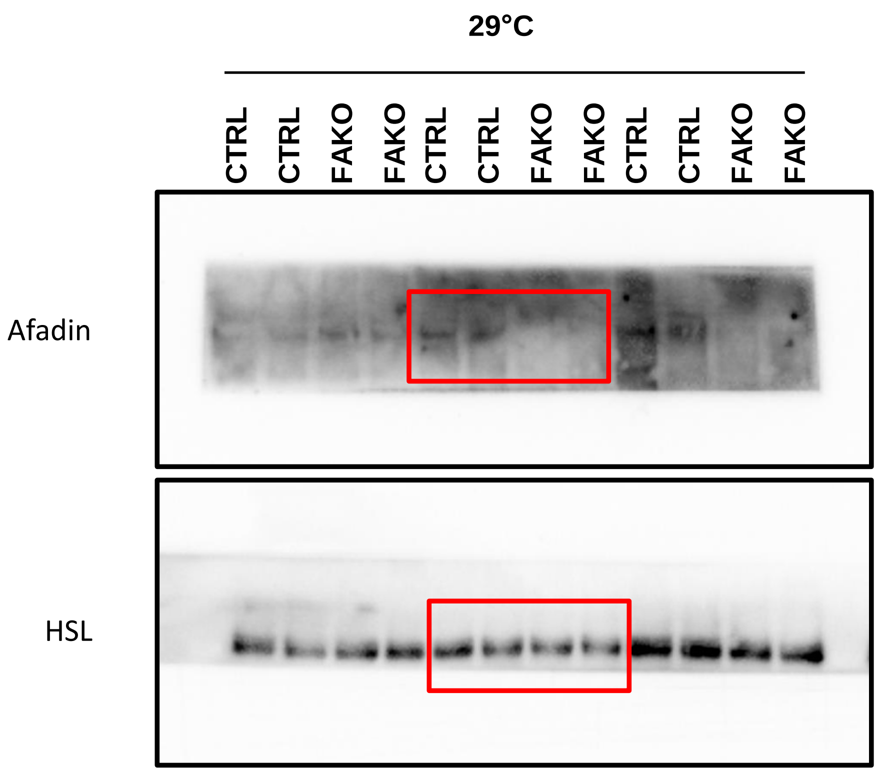

Supplement: Supplementary file 2 — Supplementary Information 2. [file 41598_2021_89207_MOESM2_ESM.pdf]
